# Impact of epicardial adipose tissue on myocardial function and structure in patients with severe aortic valve stenosis

**DOI:** 10.1002/ehf2.15422

**Published:** 2025-09-25

**Authors:** Judith Gronwald, Torben Lange, Sören J. Backhaus, Bo E. Beuthner, Ruben Evertz, Miriam Puls, Johannes T. Kowallick, Karl Toischer, Gerd Hasenfuß, Andreas Schuster, Alexander Schulz

**Affiliations:** ^1^ Department of Cardiology and Pneumology University Medical Center Göttingen, Georg‐August University and German Center for Cardiovascular Research (DZHK), Partner Site Lower‐Saxony Göttingen Germany; ^2^ Department of Cardiology Campus Kerckhoff of the Justus‐Liebig‐University Giessen, Kerckhoff Heart Centre and German Center for Cardiovascular Research (DZHK), Partner Site Rhine‐Main Bad Nauheim Germany; ^3^ Department of Cardiology and Angiology, Medical Clinic I University Hospital Giessen, Justus‐Liebig‐University Giessen Giessen Germany; ^4^ FORUM Medizin GbR Rosdorf Germany; ^5^ School of Biomedical Engineering and Imaging Sciences King's College London London UK; ^6^ Department of Medicine, Cardiovascular Division Beth Israel Deaconess Medical Center and Harvard Medical School Boston Massachusetts USA

**Keywords:** Aortic valve stenosis, cardiovascular magnetic resonance imaging, epicardial adipose tissue, left atrial function, transcatheter aortic valve replacement

## Abstract

**Aims:**

Epicardial adipose tissue (EAT) is closely associated with the development of heart failure and adverse myocardial remodelling. In patients with severe aortic valve stenosis (AS) undergoing transcatheter aortic valve replacement (TAVR), increased EAT has been identified as a predictor of adverse outcomes; however, the underlying pathophysiological mechanisms remain unclear. This study aims to explore the effects of increased EAT volumes on myocardial remodelling and dysfunction in patients with severe AS.

**Methods and results:**

One hundred thirty‐seven patients with severe AS (median age 80 years, 62% male) underwent cardiac magnetic resonance imaging (CMR) prior to TAVR. Myocardial volumes and function as well as EAT volumes were quantified from CMR acquisitions. The cohort was dichotomised at the median EAT volume. Patients with increased EAT volumes above the median (≥46.5 mL/m^2^) showed impaired left atrial (LA) reservoir strain (Es) as a distinct functional feature compared with patients with lower EAT volumes (11.8% [7.6–16.7] vs. 15.0% [10.9–19.1], *P* = 0.011), while left ventricular (LV) morphology and function (all *P* ≥ 0.216), right atrial and ventricular morphology and function (all *P* ≥ 0.090), as well as tissue characteristics (all ≥ 0.229) were similar between both groups. In a subgroup analysis of the four types of severe AS, the difference was most prominent in patients with low ejection fraction high‐gradient AS. In multivariable regression analyses, EAT was independently associated with impaired LA Es, irrespective of co‐morbidities, ventricular function, tissue characteristics and functional characteristics of AS.

**Conclusions:**

In patients with severe AS, increased EAT volume is independently associated with impaired LA function but not with other features of biventricular morphology, function or tissue composition. The incremental deterioration of LA function, in addition to the afterload imposed by AS in these patients, could increase vulnerability to heart failure and may require consideration as a therapeutic target beyond TAVR.

## Graphical abstract

Epicardial adipose tissue (EAT) has been linked to adverse remodelling and outcomes in aortic valve stenosis (AS), yet mechanisms remain unclear. In 137 patients with severe AS undergoing CMR before TAVR, higher EAT (≥46.5 ml/m^2^) was associated with impaired left atrial (LA) reservoir strain, while ventricular morphology, function and tissue characteristics were unaffected. Associations were strongest in low ejection fraction, high‐gradient AS. Multivariable analysis confirmed EAT as an independent determinant of LA dysfunction, highlighting a potential therapeutic target beyond TAVR.

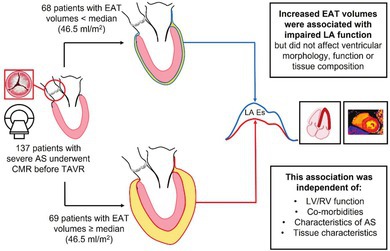

## Introduction

Severe aortic valve stenosis (AS) is the most common primary valve lesion in North America and Europe and is treated via surgical or transcatheter aortic valve replacement (TAVR).^1^ As a result of an aging population, an increasing prevalence of severe AS has been observed and is expected to further rise in the future.^2^, ^3^, ^4^ While TAVR has been the option of choice only for patients at high risk for surgical treatment in the past years, recent studies have now shown non‐inferiority to surgery in younger patients and patients with low to intermediate risk profile.^5^, ^6^, ^7^

The demographic shift towards younger patients at lower risk receiving TAVR has led to an increased focus on the long‐term prognosis of this procedure. Despite advances in the management of AS patients, heart failure remains the leading cause of rehospitalisation after TAVR and is associated with significantly worse outcomes.^8^ Consequently, factors influencing myocardial dysfunction and prognosis beyond AS, which were previously overlooked, are now coming into focus.^9^ Identifying and addressing these factors is crucial for optimising patient management and improving outcomes.

In this context, epicardial adipose tissue (EAT) is a significant and perhaps underestimated element, in addition to conventional risk factors. EAT is a type of visceral fat located between the myocardial surface and the visceral layer of the pericardium.^10^ In physiological conditions, EAT provides energy to the myocardium by releasing abundant free fatty acids as well as acting as a buffering and thermoregulatory system.^11^, ^12^ However, there are certain pathological conditions that lead to a transformation of the EAT with subsequent negative effects on the surrounding tissue, including proinflammatory and oxidative stimuli^12^, ^13^ as well as a mechanically restrictive component.^14^, ^15^ Previous studies confirmed that these stimuli of EAT promote adverse myocardial remodelling and cardiac dysfunction in patients with atrial cardiomyopathy^16^ via fibro‐fatty infiltration, resulting in heart failure progression, and contribute to coronary artery disease.^17^

In patients with severe AS undergoing TAVR, higher volumes of EAT were most recently associated with adverse outcomes and EAT was even shown to be an independent predictor for worse long‐term outcomes of affected patients.^18^ While this association is crucial for improved long‐term risk stratification, there remains a paucity of the exact pathomechanistic link between EAT and cardiac morphological and functional remodelling in this patient group.

Cardiac magnetic resonance imaging (CMR) is a modality well‐suited to study this relationship, leveraging techniques for non‐invasive EAT‐quantification,^19^ comprehensive quantification of biatrial and ventricular volumes and function, as well as describing myocardial tissue composition within a single scan.

This study aims to identify distinct characteristics and differences in myocardial tissue composition, as well as functional and structural remodelling, in patients with severe AS scheduled for TAVR with increased EAT volumes using comprehensive CMR. An improved understanding of the pathomechanistic impact of EAT on myocardial remodelling and dysfunction may improve a more personalised approach to patient management.

## Methods

### Patient cohort

Between January 2017 and March 2022, patients with symptomatic severe AS scheduled for TAVR were prospectively enrolled for systematic evaluation as part of an interdisciplinary research project at the University Medical Center Göttingen.^20^ Severe AS was diagnosed according to current guideline recommendations,^4^ using transthoracic echocardiography (Philips ie33 or Philips Epic7 [Philips Healthcare, Eindhoven, The Netherlands]). All echocardiograms performed during clinical routine were retrospectively reviewed by a single observer, using Q‐Station 3.8.5 (Philips Healthcare, Eindhoven, The Netherlands). While 62 patients (45%) had a normal ejection fraction (EF) high‐gradient AS (type I), 23 patients (17%) had a low EF high‐gradient AS (type II), 20 patients (15%) had a low EF low‐gradient AS (type III) and 32 patients (23%) had a paradoxical low‐flow low‐gradient AS (type IV). Patient characteristics, including cardiovascular risk factors, as well as medical history and laboratory markers, were systematically evaluated during follow‐up visits. In addition, results of the Minnesota LIVING WITH HEART FAILURE® (MLHFQ) questionnaire, 6‐min walk test (6mwt) and New York Heart Association (NYHA) class were documented to assess quality of life and functional capacity. All patients provided written informed consent prior to participation. The study was conducted in accordance with the Declaration of Helsinki and approved by the local ethics committee (reference number 10/5/16).

### Cardiovascular magnetic resonance imaging

CMR images were obtained on a 3.0 T MR scanner (MAGNETOM Skyra, Siemens Healthcare, Erlangen, Germany) using a 32‐channel cardiac surface receiver coil. Volumetric measures were performed on balanced steady‐state free precession cine‐sequence (bSSFP) short‐axis (SAX) stacks. Typical imaging parameters were as follows: 25 frames per cardiac cycle, time of echo (TE) 1.5 ms, time of repetition (TR) 55 ms, flip angle 55°, slice thickness 7 mm with a 7.7 mm inter‐slice gap.

T1‐mapping was performed before and 20 min after admission of a 0.15 mmol/kg bodyweight gadolinium bolus, using conventional 5(3)3 Modified Look‐Locker Inversion Recovery sequences (field of view: 360 × 360mm^2^, in‐plane resolution 1.41 × 1.41 × 8mm^3^, TR 280 ms, TE 1.12 ms, TI 180 ms, flip angle 35°, bandwidth 1085 Hz/pixel with total acquisition of 11 heart beats).^21^

### Image analysis

Commercially available software was used for post‐processing (QMass®, version 4.0.56, Medis Medical Imaging Systems, Leiden, The Netherlands). Volumetric measures of the left ventricle (LV) and right ventricle (RV) were obtained by manually delineating epi‐ and endocardial borders in SAX stacks covering the ventricles from base to apex. All volumetric measurements were indexed to body surface area.^22^

**Figure 1:**
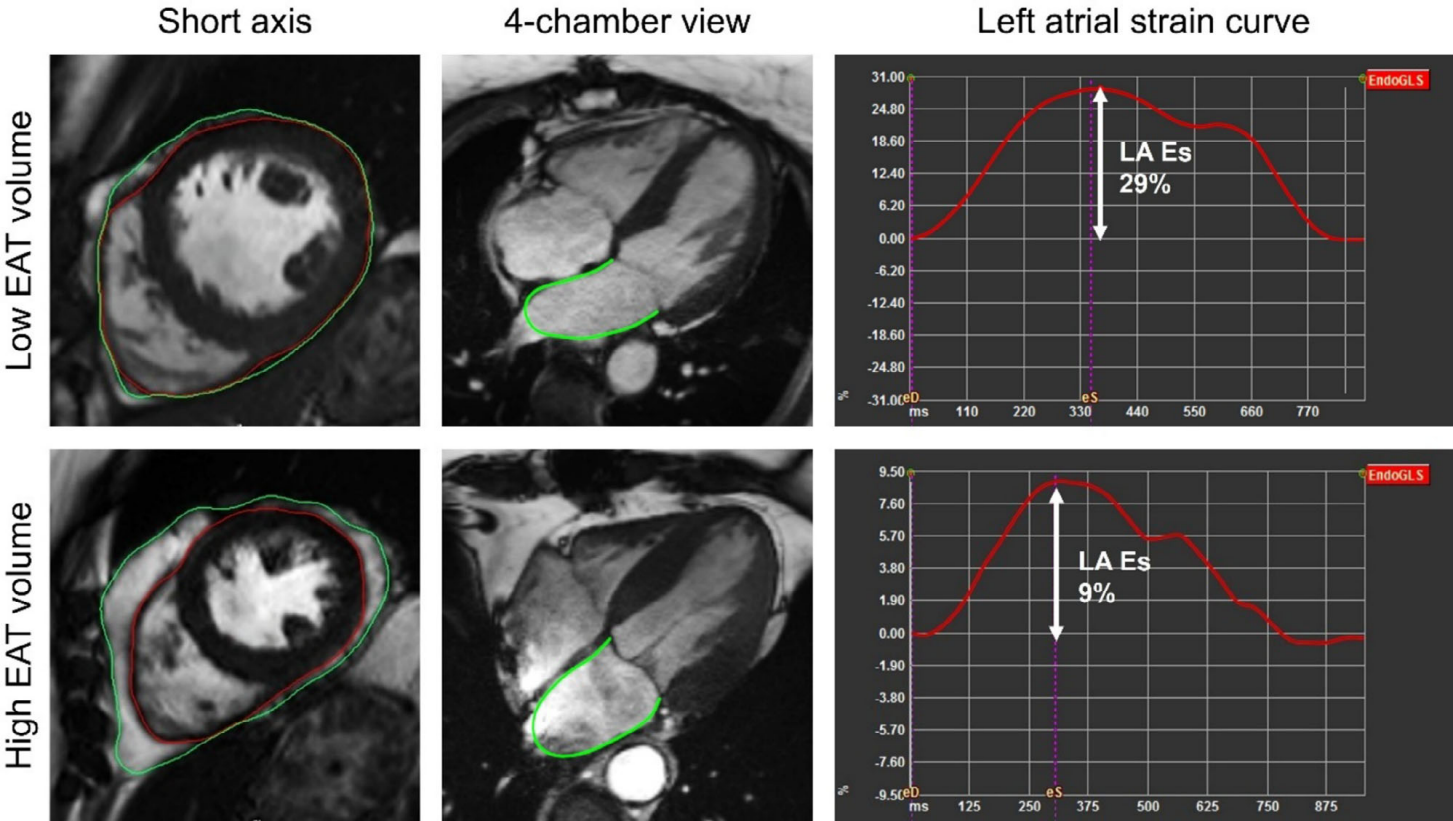
Quantification of epicardial adipose tissue und left atrial strain measurement. Manual quantification of epicardial adipose tissue (EAT) volume (left column) using short‐axis stacks, the EAT is located between the green and the red line. The middle column shows an example of left atrial (LA) strain measurement using the 4‐chamber view; after expanding the measurement to the 2‐chamber view (not shown here), strain curves as shown in the right column are obtained. The top row shows a patient with low EAT volume and high LA strain, while the bottom row shows a patient with high EAT volume and low LA strain. LA Es, left atrial reservoir strain.

CMR‐feature‐tracking‐derived ventricular strain analyses were obtained from bSSFP images (QStrain®, version 4.0.4.8, Medis Medical Imaging Systems, Leiden, The Netherlands).^23^ End‐diastolic and ‐systolic LV epi‐ and endocardial borders were manually traced. LV global longitudinal strain (GLS) was measured in 2‐, 3‐ and 4‐chamber long‐axis views, while RV GLS was obtained from 4‐chamber views.^24^ For left atrial (LA) strain analysis, LA endocardial contours were delineated in 2‐ and 4‐chamber views (compare *Figure* *1*) and reported as LA reservoir (LA Es), conduit (LA Ee) and booster pump (LA Ea) strain,^25^ right atrial (RA) strain was measured using 4‐chamber views.

For quantitative T1 and extracellular volume (ECV) mapping, QMap Software (version 2.2.36, Medis Medical Imaging Systems, Leiden, The Netherlands) was used. High‐risk remodelling index was defined as ECV > 29.1%, as proposed by Everett et al.^26^

Late gadolinium enhancement (LGE) was quantified on SAX images of inversion recovery sequences contouring epi‐ and endocardial borders and setting a 3SD threshold of signal intensity for detection of LGE in % of LV volume.^27^ Focal LGE areas were excluded from ECV analysis. To calculate LV matrix volume, total LV myocardial volumes were multiplied by ECV(%). Similarly, LV cellular volume was obtained by multiplying LV myocardial volumes by (1 − ECV[%]).^28^

### Quantification of epicardial adipose tissue

Prior to EAT quantification, the presence of EAT was validated using pre‐ and post‐contrast T1 maps, comparing T1 times of subcutaneous fat with those of suspected EAT. EAT was defined as the adipose tissue between the outer myocardial layer and the visceral pericardial layer. Ventricular EAT was manually delineated on end‐diastolic SAX slices (*Figure* *1*), from the most basal slice (indicated by the mitral valve annulus position) to the most apical slice of the ventricle.^19^ The modified Simpson's rule was used to calculate the amount of EAT.^29^ EAT quantification was conducted following an established and standardised protocol by a trained observer who was blinded to clinical and echocardiographic characteristics (J.G., 3 years of experience in CMR). For further evaluations, the study cohort was dichotomised at the median of the EAT volume.

### Statistical analysis

All statistical analyses were conducted with SPSS Statistics 29.0.0.0 (IBM, Armonk, New York, USA) and GraphPad Prism 8.0.2 (GraphPad Software, California, USA). Continuous variables are displayed as median with interquartile ranges and were compared using the nonparametric Mann–Whitney *U* or Kruskal‐Wallis test. Categorical data are presented as frequencies with corresponding percentages and were compared using the chi‐square test. After identification of distinctive morphological and functional features in between patients with high and low EAT, univariable linear regression analyses were performed to investigate the relationship between EAT volumes, co‐morbidities and other functional and morphological parameters with these features. All parameters were adjusted for age, sex and body mass index (BMI). Furthermore, parameters significantly associated with the distinctive feature were tested in four multivariable regression models. The first model included co‐morbidities, the second group echocardiographic parameters characterising the AS, the third model included CMR‐derived measurements of ventricular dimensions and function, and the last model included myocardial tissue characteristics. All *P*‐values are reported two‐sided and considered statistically significant at an alpha level below 0.05.

## Results

### Patient cohort

**Figure 2:**
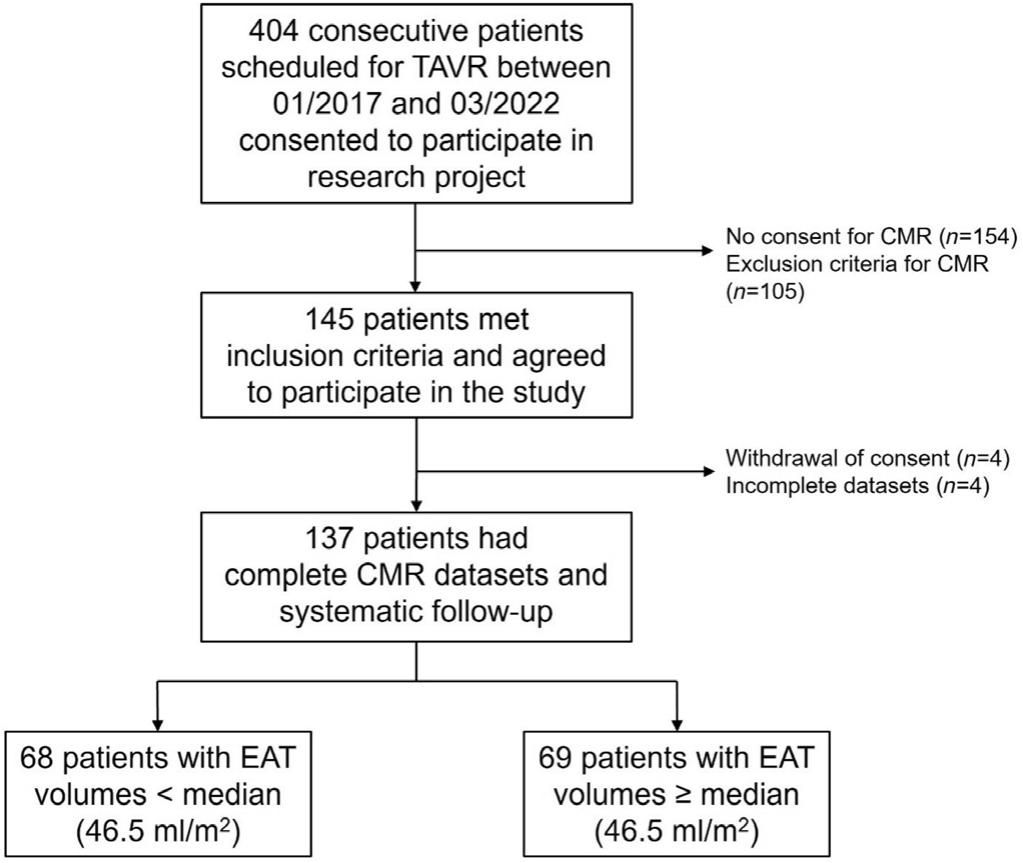
Study flowchart. CMR, cardiovascular magnetic resonance imaging; EAT, epicardial adipose tissue.

As displayed in *Figure* *2*, 404 consecutive patients were scheduled for TAVR and enrolled for systematic assessments,^30^ of which 105 patients fulfilled exclusion criteria for CMR and 154 patients refused to undergo an additional CMR after informed consent. After withdrawal of consent (*n* = 4) and exclusion due to low image quality (*n* = 4), the final study cohort consisted of 137 patients.

### Baseline characteristics

The median age of the study cohort was 80 (74–83) years, and 62% of the patients were male. The characteristics of the AS in the overall cohort were as follows: maximum velocity 4.1 m/s (3.6–4.4), mean pressure gradient 38.0 mmHg (29.0–47.8), aortic valve opening area 0.7cm^2^ (0.6–0.8) and LVEF 55% (45–59).

As outlined in *Table* *1* and *Figure* *2*, the cohort was dichotomised at the median of the total EAT volume (46.5 mL/m^2^) into a group with high and low EAT volumes. The group with higher EAT volumes had a greater proportion of male patients (36 [53%] vs. 50 [72%], *P* = 0.018) but was of comparable age. No differences were seen in BMI, co‐morbidities and echocardiographic characteristics of AS. There was no difference in quality of life (MHLFQ 25.0 [17.0–38.0] vs. 20.5 [10.3–40.5], *P* = 0.925), and functional capacity (six‐minute walk distance 307.0 m [134.8–393.8] vs. 295.0 m [180.5–376.0], *P* = 0.948) between both groups. Patients with high and low EAT volumes had comparable NYHA classes (1 [1%]/30 [44%]/36 [53%]/1 [1%] vs. 4 [6%]/19 [28%]/43 [62%]/2 [3%], *P* = 0.156) and NT‐proBNP levels (1126.5 ng/L [235.0–3341.8] vs. 1200.0 ng/L [541.3–3070.5], *P* = 0.803).

**Table 1:** Baseline characteristics

Parameter	Low EAT volumes (*n* = 68)	High EAT volumes (*n* = 69)	*P*‐value
Patient characteristics and laboratory markers			
Age at CMR (years)	81.0 (75.0–83.0)	78.5 (73.3–83.0)	0.084
Sex: female/male (*n*; %)	32 (47)/36 (53)	19 (28)/50 (72)	0.018*
BMI (kg/m^2^)	26.4 (24.2–29.7)	28.5 (24.9–30.8)	0.214
NYHA class: I/II/III/IV (*n*; %)	1(1)/30(44)/36(53)/1(1)	4(6)/19(28)/43(62)/2(3)	0.156
EuroScore II (%)	2.4 (1.8–3.4)	2.8 (1.8–6.5)	0.788
6mwt distance (m)	307.0 (134.8–393.8)	295.0 (180.5–376.0)	0.948
MLHFQ (points)	25.0 (17.0–38.0)	20.5 (10.3–40.5)	0.925
NT‐proBNP (ng/L)	1126.5 (235.0–3341.8)	1200.0 (541.3–3070.5)	0.803
Creatinine (mg/dL)	1.0 (0.9–1.2)	1.1 (0.9–1.3)	0.211
Medical history			
Arterial hypertension (*n*; %)	57 (84)	58 (84)	0.654
Diabetes (*n*; %)	19 (28)	25 (36)	0.245
Hyperlipoproteinemia (*n*; %)	42 (62)	47 (68)	0.493
Prior stroke/TIA (*n*; %)	9 (13)	9 (13)	0.973
Atrial fibrillation (*n*; %)	17 (25)	26 (38)	0.085
Coronary artery disease (*n*; %)	15 (22)	19 (28)	0.458
Prior myocardial infarction (*n*; %)	6 (9)	8 (12)	0.592
COPD (*n*; %)	5 (7)	7 (10)	0.581
Echocardiographic characteristics of the aortic valve stenosis			
Ejection fraction Simpson (%)	55.8 (48.7–61.4)	54.0 (39.8–58.0)	0.377
Stroke volume index (mL/m^2^)	34.9 (27.9–44.1)	37.0 (26.4–44.8)	0.481
Maximum velocity (m/s)	4.1 (3.6–4.4)	4.1 (3.6–4.5)	0.557
Mean pressure gradient (mmHg)	40.0 (31.0–49.0)	38.5 (30.5–47.8)	0.826
Aortic valve opening area (cm^2^)	0.7 (0.5–0.8)	0.7 (0.6–0.9)	0.354

*Note:* Analysis of baseline characteristics after dichotomisation at the median of EAT at 46.5 mL/m^2^. The asterisk indicates statistical significance.

6mwt, 6‐min walk test; BMI, body mass index; COPD, chronic obstructive lung disease; EAT, epicardial adipose tissue; MLHFQ, Minnesota LIVING WITH HEART FAILURE® questionnaire; NYHA, New York Heart Association; TIA, transient ischaemic attack.

### CMR‐derived functional and morphological differences in patients with high and low epicardial adipose tissue volumes

**Figure 3:**
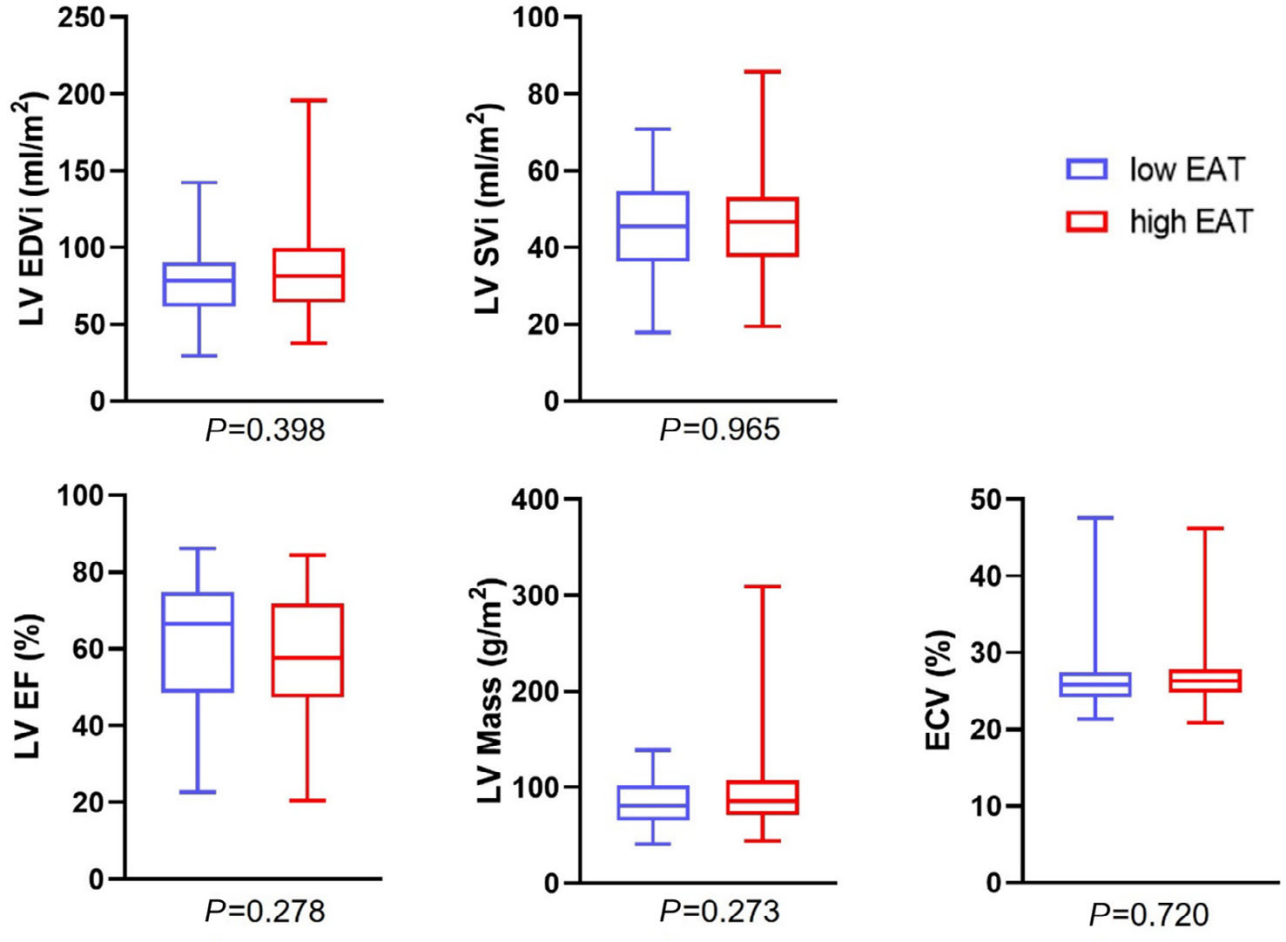
Left ventricular dimensions, function and extra cellular volume depending on epicardial adipose tissue volumes. After dichotomisation at the median total EAT volume of 46.5 mL/m^2^, both patient groups showed comparable left ventricular volumes and function. Box plots show minimum, maximum and median with interquartile range. EAT, epicardial adipose tissue; ECV, extracellular volume; EDVi, end‐diastolic volume index; EF, ejection fraction; LV, left ventricle; SVi, stroke volume index.

**Figure 4:**
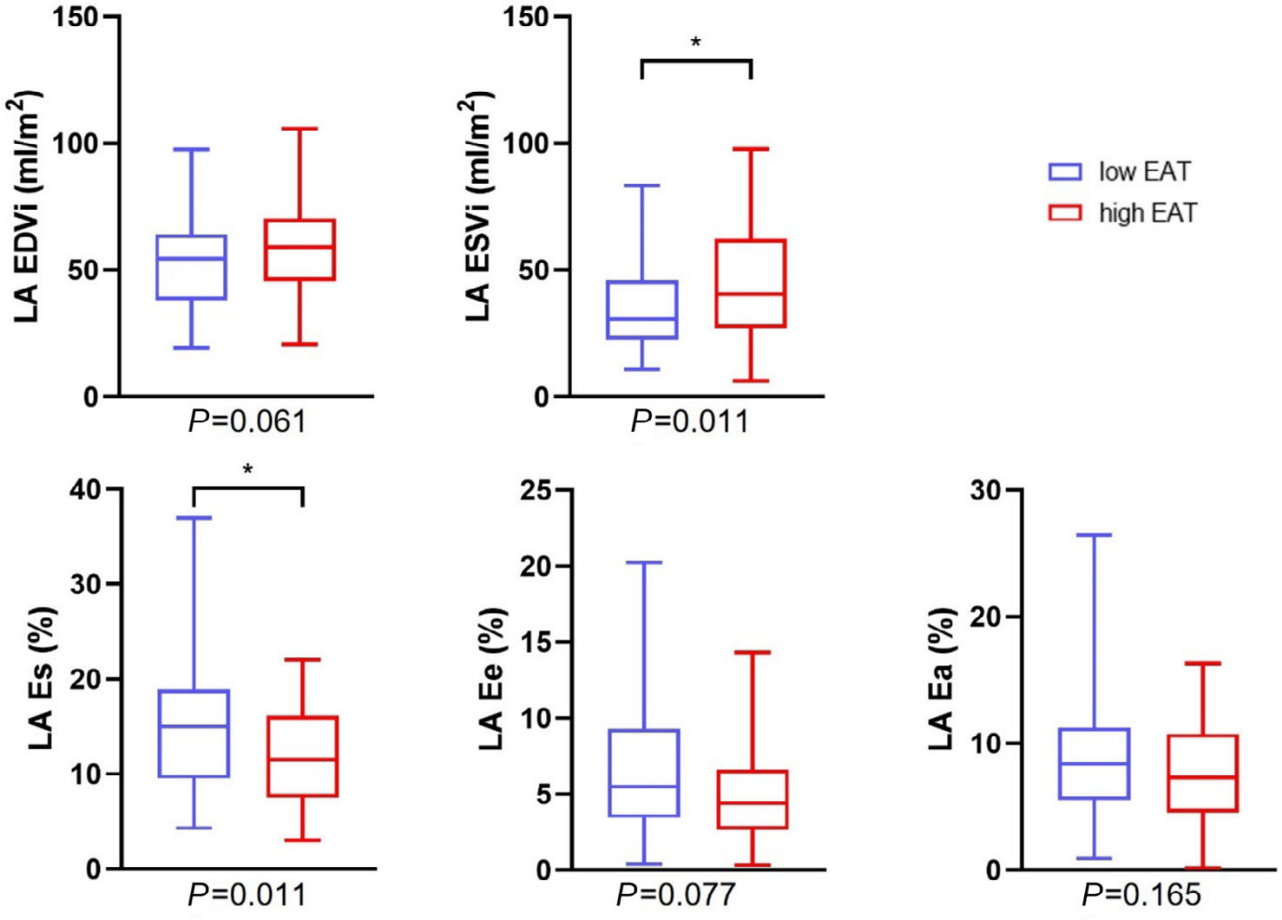
Left atrial dimensions and function depending on epicardial adipose tissue volumes. Patients with total EAT volumes ≥ 46.5 mL/m^2^ showed a higher LA ESVi and an impaired LA Es compared with patients with lower total EAT volumes while EDVi, LA Ee and LA Ea were not significantly different between the two groups. Box plots show minimum, maximum and median with interquartile range. The asterisk indicates statistical significance. Ea, booster pump strain; EAT, epicardial adipose tissue; EDVi, end‐diastolic volume index; Ee, conduit strain; Es, reservoir strain; ESVi, end‐systolic volume index; LA, left atrium.

As shown in *Table* *2* and *Figure* *3*, patients with low and high EAT volumes had comparable LV and RV dimensions and function (all *P* ≥ 0.273). Morphological and functional analyses of the atria revealed increased LA end‐systolic volumes (29.4 mL/m^2^ [19.7–39.4] vs. 40.9 mL/m^2^ [30.2–59.3], *P* = 0.011) and a lower LA stroke volume index (15.8 mL/m^2^ [12.4–23.0] vs. 14.2 mL/m^2^ [8.5–21.5], *P* = 0.030) in patients with high EAT volumes, while end‐diastolic volumes did not differ. Concordantly, LA Es was lower in patients with high EAT volumes compared with patients with low EAT volumes (15.0% [10.9–19.1] vs. 11.8% [7.6–16.7], *P* = 0.011). LA conduit strain also showed a trend towards lower values in patients with increased EAT volumes (5.6% [3.5–9.3] vs. 4.0% [2.6–6.4], *P* = 0.077); meanwhile, there was no difference in LA booster pump strain (8.4% [5.4–11.4] vs. 7.2% [4.6–10.8], *P* = 0.165), as shown in *Figure* *4*. No difference in RA morphology and function could be observed between both groups.

**Table 2:** Cardiovascular magnetic resonance parameters

Parameter	Low EAT (*n* = 68)	High EAT (*n* = 69)	*P*‐value
Left ventricle			
LVEF (%)	67.1 (56.4–74.4)	57.8 (46.6–76.8)	0.378
LV mass (g/m^2^)	78.7 (63.8–94.3)	88.1 (65.5–108.2)	0.216
LV EDVi (mL/m^2^)	73.3 (65.6–108.2)	80.9 (64.1–101.2)	0.328
LV ESVi (mL/m^2^)	21.4 (16.2–34.8)	34.4 (18.4–55.2)	0.268
LV SVi (mL/m^2^)	45.7 (36.6–54.7)	46.2 (36.3–53.0)	0.965
LV GLS (%)	−23.1 (−27.9—17.8)	−19.9 (−26.0—15.2)	0.100
LV GCS (%)	−40.2 (−47.9—32.8)	−36.0 (−44.8—26.9)	0.244
LV GRS (%)	67.8 (47.9–89.7)	59.9 (43.2–74.0)	0.062
Right ventricle			
RVEF (%)	54.2 (45.5–61.5)	50.8 (43.3–57.8)	0.523
RV mass (mL/m^2^)	20.3 (17.5–23.9)	19.0 (13.1–23.7)	0.637
RV EDVi (mL/m^2^)	67.1 (59.6–80.7)	69.1 (59.6–79.0)	0.755
RV ESVi (mL/m^2^)	28.2 (23.4–37.2)	32.4 (23.5–45.9)	0.745
RV SVi (mL/m^2^)	37.3 (28.8–43.4)	34.7 (27.2–38.4)	0.324
RV GLS (%)	−26.3 (−31.1—21.6)	−25.8 (−32.7—21.6)	0.885
Left atrium			
LA EDVi (mL/m^2^)	53.3 (37.7–64.1)	58.8 (45.4–69.9)	0.061
LA ESVi (mL/m^2^)	29.1 (22.3–42.9)	37.8 (26.8–61.2)	0.011*
LA SVi (mL/m^2^)	15.8 (12.6–23.2)	14.7 (8.8–21.5)	0.030*
LA Es (%)	15.0 (10.9–19.1)	11.8 (7.6–16.7)	0.011*
LA Ee (%)	5.6 (3.5–9.3)	4.0 (2.6–6.4)	0.077
LA Ea (%)	8.4 (5.4–11.4)	7.2 (4.6–10.8)	0.165
Right atrium			
RA EDVi (mL/m^2^)	46.4 (29.7–57.4)	49.5 (37.6–73.2)	0.363
RA ESVi (mL/m^2^)	30.6 (25.6–39.3)	32.8 (23.7–55.1)	0.601
RA SVi (mL/m^2^)	12.3 (8.1–17.2)	11.9 (8.0–16.0)	0.755
RA Es (%)	15.8 (11.3–21.6)	14.5 (8.2–19.9)	0.191
RA Ee (%)	6.8 (3.5–9.5)	5.6 (2.5–9.5)	0.721
RA Ea (%)	9.7 (7.0–12.1)	7.9 (5.1–11.0)	0.090
Tissue characterisation			
Total LGE (3SD) (mL/m^2^)	9.1 (4.4–13.2)	6.9 (4.7–13.4)	0.386
T1 native septal (ms)	1309 (1276–1323)	1320 (1281–1356)	0.420
ECV total (%)	26.6 (24.7–29.6)	27.0 (25.5–28.7)	0.720
ECV corrected (%)	26.1 (24.7–27.3)	26.9 (25.2–28.2)	0.229
ECV corrected > 29.1% (*n*; %)	6 (15)	8 (17)	0.871
LV matrix volume (mL/m^2^)	17.8 (13.7–20.6)	19.9 (15.8–24.7)	0.257
LV cellular volume (mL/m^2^)	48.9 (37.5–62.8)	51.1 (42.4–67.8)	0.392

Analysis of CMR‐derived parameters after dichotomisation at the median of EAT at 46.5 mL/m^2^. Quantitative data are presented as median with interquartile ranges. Qualitative data are presented as *n* (%). The Mann–Whitney *U* test was used to test for significant differences. The asterisk indicates statistical significance.

Ea, booster pump strain; EAT, epicardial adipose tissue; ECV, extra cellular volume; EDVi, end‐diastolic volume index; Ee, conduit strain; Es, reservoir strain; ESVi, end‐systolic volume index; GCS, global circumferential strain; GLS, global longitudinal strain; GRS, global radial strain; LA, left atrium; LGE, late gadolinium enhancement; LV, left ventricle; LVEF, left ventricular ejection fraction; RA, right atrium; SVi, stroke volume index.

### Impact of epicardial adipose tissue on myocardial tissue composition in patients with severe aortic valve stenosis

As highlighted in *Table* *2*, T1 times and ECV of patients with low EAT volumes were not different compared with patients with high EAT volumes (T1 time: 1309 ms [1276–1323] vs. 1320 ms [1281–1356], *P* = 0.420; ECV: 26.6% [24.7–29.6] vs. 27.0% [25.5–28.7], *P* = 0.720). Concordantly, LV matrix and cellular volumes were not different between both groups (*P* ≥ 0.257). Both groups had similar proportions of patients having a high‐risk remodelling index (6 [15%] vs. 8 [17%], *P* = 0.871). Moreover, total LGE volumes did not differ between the two groups (9.1 mL/m^2^ [4.4–13.2] vs. 6.9 mL/m^2^ [4.7–13.4], *P* = 0.386).

### Impact of epicardial adipose tissue on different subgroups of severe aortic valve stenosis

Tables S1–S4 detail subgroup analyses of patients with low and high EAT volumes in all four types of AS. In AS type II, RA end‐diastolic volumes were increased in patients with high EAT volumes compared with patients with low EAT volumes (100.7 mL/m^2^ [72.1–110.7] vs. 44.7 mL/m^2^ [35.6–53.8], *P* = 0.022). Moreover, LA function was significantly impaired in patients with high EAT volumes, including LA stroke volume (9.3 mL/m^2^ [3.8–24.4] vs. 22.0 mL/m^2^ [15.6–28.0], *P* = 0.026), LA reservoir (8.2% [4.8–13.5] vs. 16.6% [13.1–18.8], *P* = 0.008), conduit (3.4% [2.2–5.6] vs. 5.5% [4.5–9.4], *P* = 0.026) and booster pump strain (5.2% [3.1–7.5] vs. 9.4% [7.7–12.1], *P* = 0.008). Additionally, LV GLS was impaired in patients with high EAT volumes in this subgroup (−17.5% [−18.8—14.8] vs. −13.5% [−17.1—9.9], *P* = 0.047). Within the other AS subgroups, no statistical difference was observed for functional and morphological cardiac characteristics between patients with high and low EAT volumes. However, LA Es showed a numerical trend towards an impaired LA function in patients with high EAT volumes compared with patients with low EAT volumes in all AS subgroups except for type III AS, where patients with low and high EAT volumes had similar LA Es (10.9% [8.0–19.0] vs. 11.0% [9.0–18.6], *P* = 0.683).

### Association of epicardial adipose tissue volumes with impaired left atrial function

Impaired LA function as measured by LA Es was identified as the main distinctive feature of patients with higher EAT volumes. As displayed in *Table* *3*, increased EAT volumes were directly associated with impaired LA Es (β = −0.227, *P* = 0.011) in univariable linear regression analyses. Beyond EAT, history of atrial fibrillation (β = −0.471, *P* < 0.001), reduced echocardiographic LVEF (β = 0.222, *P* = 0.017), impaired stroke volume index (β = 0.301, *P* < 0.001) and smaller aortic valve opening area (β = 0.189, *P* = 0.031) were associated with impaired LA Es. For CMR‐derived measurements, impaired LV GLS (β = −0.233, *P* = 0.026) and impaired RV GLS (β = −0.287, *P* = 0.002) as well as an increased LV end‐diastolic volume index (β = −0.033, *P* = 0.735) showed associations with decreasing LA Es.

**Table 3:** Multivariable regression analysis

	LA Es univariable regression analysis		LA Es multivariable regression analysis	
	Coefficient *β*	*P*‐value	Standardised coefficient *β*	*P*‐value
1. Co‐morbidities				
EAT (mL/m^2^)	−0.227	0.011*	−0.172	0.034*
Diabetes	−0.082	0.370		
Arterial hypertension	0.041	0.654		
Atrial fibrillation	−0.471	<0.001*	−0.442	<0.001*
Hyperlipoproteinemia	0.139	0.124		
Prior myocardial infarction	−0.017	0.844		
Creatinine	−0.092	0.329		
2. Echocardiographic characteristics of AS				
EAT (mL/m^2^)	−0.227	0.011*	−0.199	0.022*
Ejection fraction Simpson (%)	0.222	0.017*	0.108	0.245
Maximum velocity (m/s)	0.158	0.072		
Mean pressure gradient (mmHg)	0.150	0.085		
Stroke volume index (mL/m^2^ BSA)	0.301	<0.001*	0.237	0.021*
Aortic valve opening area (cm^2^)	0.189	0.031*	0.049	0.620
3. CMR‐based ventricular morphology and function				
EAT (mL/m^2^)	−0.227	0.011*	−0.205	0.015*
LV mass (g/m^2^)	−0.129	0.170		
LV end‐diastolic volume index (mL/m^2^)	−0.231	0.016*	−0.033	0.735
LV GLS (%)	−0.400	<0.001*	−0.233	0.026*
RV GLS (%)	−0.374	<0.001*	−0.287	0.002*
4. CMR‐based tissue characteristics				
EAT (mL/m^2^)	−0.227	0.011*	−0.227	0.045*
ECV total (%)	−0.102	0.345		
ECV corrected (%)	−0.200	0.060		
T1 native septal (ms)	−0.238	0.007*	−0.232	0.029*
Total LGE (3SD) (mL/m^2^)	−0.005	0.959		
LV matrix volume (mL/m^2^)	−0.185	0.050		
LV cellular volume (mL/m^2^)	−0.129	0.185		

Multivariable regression analysis of EAT volumes with four different models: co‐morbidities, echocardiographic characteristics of the aortic valve stenosis, CMR‐based parameters of LV morphology and function, and tissue characteristics. Each value has been adjusted for age, sex, and body mass index. The asterisk indicates statistical significance.

AS, aortic valve stenosis; CMR, cardiovascular magnetic resonance; EAT, epicardial adipose tissue; LA Es, left atrial reservoir strain; LGE, late gadolinium enhancement; LV, left ventricle; RV, right ventricle.

In four models of multivariable regression analyses, increased EAT volumes remained associated with lower LA Es independent of cardiovascular risk factors and co‐morbidities (standardised β = −0.172, *P* = 0.034), echocardiographic characteristics of AS (standardised β = −0.199, *P* = 0.022), myocardial function and morphology (standardised β = −0.205, *P* = 0.015), and tissue characteristics (standardised β = −0.227, *P* = 0.045).

## Discussion

This study provides a comprehensive evaluation of the impact of EAT on myocardial morphology and function in patients with severe AS scheduled for TAVR. We identified impaired LA function as a distinctive feature of patients with increased EAT volumes while there were no differences in biventricular function and morphology as well as LV tissue composition as measured by CMR. Importantly, LA functional impairment was independently associated with higher EAT volumes irrespective of other influential parameters. Increased amounts of EAT may cause additional functional distress to affected patients, beyond adverse haemodynamic loading imposed by severe AS.

In this study, increased EAT volumes were associated with impaired LA function in patients with severe AS. While no previous studies have specifically investigated the pathomechanisms of EAT in this patient population, earlier research on other cardiovascular diseases has demonstrated that increased EAT volumes can contribute to various remodelling processes.^16^, ^17^, ^31^, ^32^ Notably, the association between EAT and LA remodelling has been consistently observed across studies. For instance, in patients with atrial fibrillation, fibro‐fatty infiltration of the myocardium was identified as a proarrhythmogenic substrate.^16^ Similarly, the Framingham Heart Study revealed a link between EAT and LA morphology,^33^ and patients with heart failure with preserved ejection fraction (HFpEF) and increased EAT volumes were shown to have significant atrial functional impairment as well.^32^ These findings align with the results of our study, highlighting the susceptibility of the LA to the influence of EAT. The observed atrial failure may contribute to reduced LV filling, thereby promoting heart failure progression and poorer clinical outcomes.^34^, ^35^

Apart from changes in LA morphology and function in patients with increased EAT volumes, a study in a HFpEF cohort showed that increased EAT volumes may also alter tissue composition and induce fibrotic remodelling as measured by prolonged T1 times.^32^ But also, ventricular structural remodelling can be driven by increased EAT volumes as shown for LV mass in a cohort with coronary artery disease^17^ and healthy subjects.^36^ In contrast, no differences in function or morphology were observed beyond the LA in the overall cohort of the present study. A possible explanation might be that the remodelling processes induced by the haemodynamic challenges of AS on ventricular structural and functional remodelling conceal the influence of EAT on LV remodelling.

To further explore the impact of EAT in the context of the hemodynamic challenges imposed by AS, we conducted subgroup analyses across different AS subtypes. While we observed a trend towards lower LA function in patients with high EAT in all AS subtypes, except for Type III, the influence of EAT was most pronounced in Type II AS. This subgroup experiences the greatest hemodynamic challenges due to high‐gradient AS combined with reduced LV EF, which was also reflected in impaired LV GLS.

Severe AS itself contributes to the development of an atrial myopathy, as increased LV filling pressures, followed by LV hypertrophy and dysfunction adversely impact the LA. This results in elevated LA pressures, which lead to LA enlargement and remodelling.^37^

These findings suggest that the combined remodelling of the LA due to both EAT and AS has a particularly strong impact on a more vulnerable LA already challenged by the primary disease of severe AS. While it can be hypothesized that hemodynamically driven remodelling exerts a greater influence than EAT‐induced remodelling, our study provides evidence that EAT remodelling is independent of the severity of AS, ventricular function and co‐morbidities, establishing it as an independent pathological factor in these patients.

However, the small size of the study subgroups limits the generalizability of these findings, and the exact mechanisms underlying these interactions require further investigation in dedicated cohorts.

Three distinct pathways have been identified through which EAT may influence the mentioned myocardial remodelling processes.

On the molecular basis, EAT secretes pro‐inflammatory cytokines directly affecting the adjacent myocardium due to the close anatomic relationship of the adipose tissue.^13^ In addition to this molecular component, the accumulation of EAT results in a mechanical restriction, similar to the one caused by pericardial effusion or constrictive pericarditis.^14^, ^15^ Lastly, it was found that EAT can directly infiltrate myocardial tissue^38^ as shown in patients with atrial fibrillation, representing a structural pathway by which EAT might contribute to remodelling processes.

These described mechanisms may provide possible explanations for the observed diminished LA function in patients with severe AS. Compared with the LV, the thinner atrial wall may expose the LA to greater mechanical restriction, thereby reducing its relaxation capacity and leading to diastolic atrial dysfunction.^14^, ^15^ Additionally, EAT infiltration and inflammatory responses in the myocardium may further contribute to this process, as fatty infiltrations can impair LA myocardial integrity and function.^38^, ^39^ It may also increase susceptibility to arrhythmia, potentially leading to the development or progression of atrial myopathy.^40^ In the present cohort, this is reflected by the trend towards higher rates of atrial fibrillation in patients with elevated EAT volumes. However, these specific pathological pathways remain speculative based on the available data and warrant further investigation.

The findings of this study yield several potential implications. Previous studies have shown that both LA function and EAT have an impact on the long‐term outcome of patients with severe AS undergoing TAVR.^18^, ^41^, ^42^ Our results now suggest that there may be a direct pathomechanistic link between these two components. Therefore, EAT may emerge as an additional target to influence reverse remodelling beyond valve replacement to further improve LA function and overall outcome by preventing heart failure progression in patients with severe AS scheduled for TAVR.

Previous studies investigating EAT as a therapeutic target in other contexts have already demonstrated promising results. Statin therapy has been shown to reduce EAT thickness, lower levels of EAT‐secreted inflammatory mediators, and actively decrease EAT volumes.^13^, ^43^ Similarly, sodium‐glucose cotransporter‐2 inhibitors have been found to significantly reduce EAT.^44^, ^45^ Another promising option may be tirzepatide, which not only decreases EAT but also reduces LV mass and reverses remodelling in patients with obesity‐related HFpEF.^46^

Early treatment of patients with high EAT volumes may be particularly beneficial, as it could mitigate EAT‐induced irreversible damage to the LA^37^, ^47^ and reduce the overall vulnerability of the LA before severe AS develops. However, further studies are necessary to evaluate the effects and determine the optimal timing of these therapeutic options in patients with severe AS.

## Limitations

Even though the proposed pathways render causative explanations for the observed association, the detailed mechanism of the interconnection of EAT and atrial dysfunction in patients with severe AS cannot be investigated by the means of this study, and further studies are required to verify the temporal sequence of disease development. Furthermore, the AS subgroups are small in size, and given results may not reflect differences in larger populations. Volumetric EAT assessment can only provide quantitative information, while it cannot provide qualitative information on EAT composition and inflammatory processes. Age‐ and sex‐specific norms for EAT have not yet been defined. Therefore, dichotomisation using the median is only one of many possible methods, and cut‐offs may not apply to other populations.

## Conclusions

Patients with severe AS and increased EAT volumes, as measured by CMR, exhibited impaired LA function without evidence of additional biventricular functional or structural remodelling compared with those with lower EAT volumes. Elevated EAT volumes appeared to have additional and independent effects on LA remodelling, beyond the haemodynamic and functional challenges imposed by severe AS. Thus, EAT may independently contribute to the development of heart failure in patients with severe AS, and its quantification could enhance the development of personalised preventive strategies.

## Consent

All patients provided written informed consent.

## Conflict of interest

None declared.

## Funding

This work was supported by the German Research Foundation (DFG, CRC 1002, D1).

## Supporting information


**Table S1:** Characteristics of patients with normal ejection fraction high‐gradient aortic valve stenosis (type I).
**Table S2:** Characteristics of patients with low ejection fraction high‐gradient aortic valve stenosis (type II).
**Table S3:** Characteristics of patients with low ejection fraction low‐gradient aortic valve stenosis (type III).
**Table S4:** Characteristics of patients with paradoxical low flow low‐gradient aortic valve stenosis (type IV).

## Data Availability

The data underlying this article will be shared on reasonable request to the corresponding author.
