# Supplementary material for: Impact of epicardial adipose tissue on myocardial function and structure in patients with severe aortic valve stenosis
Source: ESC Heart Fail. 2025 Sep 25;12(6):4230–41. doi: 10.1002/ehf2.15422 (PMC12719855; doi:10.1002/ehf2.15422)
Supplement: Supplementary file 1 — Table S1: Characteristics of patients with normal ejection fraction high‐gradient aortic valve stenosis (type I). Table S2: Characteristics of patients with low ejection fraction high‐gradient aortic valve stenosis (type II). Table S3: Characteristics of patients with low ejection fraction low‐gradient aortic valve stenosis (type III). Table S4: Characteristics of patients with paradoxical low flow low‐gradient aortic valve stenosis (type IV). [file EHF2-12-4230-s001.docx]

##### Supplementary Table 1: Characteristics of patients with normal ejection fraction high-gradient aortic valve stenosis (type I)

| **Parameter** | **Low EAT volumes (*n*=31)** | **High EAT volumes (*n*=31)** | ***p*-value** |
| --- | --- | --- | --- |
| **Patient characteristics** | | | |
| Age at CMR (years) | 79.0 (74.0-81.0) | 79.0 (76.3-84.3) | 0.430 |
| Sex: female/male (*n*; %) | 15(48)/16(52) | 12(39)/19(61) | 0.442 |
| BMI (kg/m2) | 28.9 (24.7-31.8) | 28.2 (24.6-31.7) | 0.272 |
| Ejection fraction Simpson (%) | 60.2 (56.1-63.7) | 59.0 (55.0-64.9) | 0.955 |
| **Left ventricle** | | | |
| LVEF (%) | 74.2 (69.9-78.3) | 75.7 (59.7-79.1) | 0.708 |
| LV Mass (g/m^2^) | 72.2 (62.9-91.4) | 98.1 (66.6-110.1) | 0.356 |
| LV EDVi (ml/m^2^) | 72.0 (57.6-83.6) | 67.9 (57.9-84.5) | 0.762 |
| LV ESVi (ml/m^2^) | 18.5 (14.7-21.8) | 16.9 (11.8-32.7) | 0.885 |
| LV SVi (ml/m^2^) | 52.5 (42.5-58.8) | 51.8 (41.7-56.7) | 0.988 |
| LV GLS (%) | -27.3 (-29.3—23.0) | -26.3 (-30.4—20.3) | 0.501 |
| LV GCS (%) | -42.5 (-53.4—38.6) | -43.7 (-49.4—38.4) | 0.717 |
| LV GRS (%) | 79.9 (63.3-91.0) | 70.7 (61.5-88.6) | 0.403 |
| **Right ventricle** | | | |
| RVEF (%) | 58.8 (54.3-65.4) | 54.6 (46.2-60.9) | 0.584 |
| RV Mass (ml/m^2^) | 21.7 (15.3-23.8) | 18.3 (16.7-20.9) | 0.209 |
| RV EDVi (ml/m^2^) | 67.4 (57.3-80.4) | 61.7 (55.6-67.6) | 0.141 |
| RV ESVi (ml/m^2^) | 26.7 (22.3-31.7) | 26.8 (22.6-31.4) | 0.574 |
| RV SVi (ml/m^2^) | 41.1 (34.8-47.1) | 34.2 (30.8-36.5) | 0.062 |
| RV GLS (%) | -29.6 (-32.0—23.5) | -31.4 (-35.0—25.6) | 0.258 |
| **Left atrium** | | | |
| LA EDVi (ml/m^2^) | 42.5 (35.7-63.1) | 50.6 (42.4-62.9) | 0.145 |
| LA ESVi (ml/m^2^) | 26.9 (19.0-35.0) | 31.1 (24.2-46.2) | 0.106 |
| LA SVi (ml/m^2^) | 19.1 (13.5-26.2) | 20.4 (15.2-23.6) | 0.554 |
| LA Es (%) | 18.5 (16.1-20.7) | 15.1 (12.3-19.6) | 0.176 |
| LA Ee (%) | 7.4 (4.1-11.2) | 5.0 (3.9-8.8) | 0.325 |
| LA Ea (%) | 10.7 (6.6-13.4) | 8.7 (6.8-11.8) | 0.641 |
| **Right atrium** | | | |
| RA EDVi (ml/m^2^) | 45.9 (29.7-56.8) | 39.1 (35.0-51.8) | 0.988 |
| RA ESVi (ml/m^2^) | 29.1 (17.8-37.0) | 25.7 (21.5-34.4) | 0.795 |
| RA SVi (ml/m^2^) | 12.3 (8.3-22.5) | 13.6 (10.4-18.6) | 0.718 |
| RA Es (%) | 19.2 (15.5-23.8) | 17.9 (15.3-25.0) | 0.355 |
| RA Ee (%) | 8.3 (4.1-14.3) | 8.9 (5.1-15.5) | 0.750 |
| RA Ea (%) | 10.6 (7.0-12.6) | 9.8 (6.2-11.2) | 0.111 |
| **Tissue characterisation** | | | |
| Total LGE (3SD) (ml/m^2^) | 8.3 (5.4-15.2) | 7.0 (4.2-11.5) | 0.545 |
| T1 native septal (ms) | 1301.6 (1266.0-1308.9) | 1307.2 (1273.1-1336.7) | 0.279 |
| ECV total (%) | 25.0 (23.9-26.5) | 26.9 (25.2-28.1) | 0.201 |
| ECV corrected (%) | 25.0 (23.2-26.5) | 26.1 (25.0-27.8) | 0.217 |
| LV matrix volume (ml/m^2^) | 15.2 (13.1-18.5) | 21.5 (15.7-26.3) | 0.147 |
| LV cellular volume (ml/m^2^) | 48.7 (39.1-52.7) | 59.8 (44.1-70.2) | 0.166 |

Analysis of patient characteristics after dichotomisation at the median of EAT at 46.5ml/m^2^. Quantitative data are presented as median with interquartile ranges. Qualitative data are presented as n (%). The Mann-Whitney U test was used to test for significant differences. The asterisk indicates statistical significance. Ea: booster pump strain, EAT: epicardial adipose tissue, ECV: extra cellular volume, EDVi: end-diastolic volume index, Ee: conduit strain, Es: reservoir strain, ESVi: end-systolic volume index, GCS: global circumferential strain, GLS: global longitudinal strain, GRS: global radial strain, LA: left atrium, LGE: late gadolinium enhancement, LV: left ventricle, LVEF: left ventricular ejection fraction, RA: right atrium, SVi: stroke volume index

##### Supplementary Table 2: Characteristics of patients with low ejection fraction high-gradient aortic valve stenosis (type II)

| **Parameter** | **Low EAT volumes (*n*=8)** | **High EAT volumes (*n*=15)** | ***p*-value** |
| --- | --- | --- | --- |
| **Patient characteristics** | | | |
| Age at CMR (years) | 88.0 (88.0-88.9) | 73.0 (67.8-79.0) | 0.038* |
| Sex: female/male (*n*; %) | 2(25)/6(75) | 2(13)/13(87) | 0.482 |
| BMI (kg/m2) | 18.5 (17.9-19.0) | 26.9 (20.8-33.8) | 0.107 |
| Ejection fraction Simpson (%) | 42.3 (42.0-42.5) | 41.7 (22.8-46.4) | 0.949 |
| **Left ventricle** | | | |
| LVEF (%) | 54.6 (50.2-59.0) | 46.0 (25.6-49.9) | 0.121 |
| LV Mass (g/m^2^) | 106.7 (81.1-132.4) | 108.0 (87.8-118.4) | 0.796 |
| LV EDVi (ml/m^2^) | 108.2 (78.3-138.2) | 101.5 (97.3-112.8) | 0.302 |
| LV ESVi (ml/m^2^) | 50.5 (32.1-68.9) | 55.4 (45.7-78.0) | 0.272 |
| LV SVi (ml/m^2^) | 57.7 (46.2-69.3) | 50.4 (37.3-52.7) | 0.519 |
| LV GLS (%) | -17.5 (-18.8—14.8) | -13.5 (-17.1—9.9) | 0.047* |
| LV (GCS (%) | -32.8 (-37.7—18.0) | -26.7 (-31.8—16.8) | 0.447 |
| LV GRS (%) | 40.4 (38.3-66.2) | 40.1 (25.4-51.1) | 0.447 |
| **Right ventricle** | | | |
| RVEF (%) | 47.2 (37.9-56.6) | 46.7 (14.7-67.3) | 0.138 |
| RV Mass (ml/m^2^) | 23.3 (15.3-31.4) | 26.1 (12.1-30.9) | 0.272 |
| RV EDVi (ml/m^2^) | 88.0 (63.7-112.3) | 79.8 (65.6-99.5) | 0.156 |
| RV ESVi (ml/m^2^) | 48.7 (27.7-69.8) | 48.7 (21.4-64.3) | 0.107 |
| RV SVi (ml/m^2^) | 39.3 (36.1-42.5) | 34.8 (11.1-44.2) | 1.0 |
| RV GLS (%) | -17.3 (-28.7—16.0) | -23.2 (-30.4—15.0) | 1.0 |
| **Left atrium** | | | |
| LA EDVi (ml/m^2^) | 58.6 (46.3-70.9) | 60.1 (56.9-79.6) | 0.307 |
| LA ESVi (ml/m^2^) | 33.1 (25.7-40.4) | 54.6 (36.6-75.8) | 0.113 |
| LA SVi (ml/m^2^) | 22.0 (15.6-28.0) | 9.3 (3.8-24.4) | 0.026* |
| LA Es (%) | 16.6 (13.1-18.8) | 8.2 (4.8-13.5) | 0.008* |
| LA Ee (%) | 5.5 (4.5-9.4) | 3.4 (2.2-5.6) | 0.026* |
| LA Ea (%) | 9.4 (7.7-12.1) | 5.2 (3.1-7.5) | 0.008* |
| **Right atrium** | | | |
| RA EDVi (ml/m^2^) | 44.7 (35.6-53.8) | 100.7 (72.1-110.7) | 0.022* |
| RA ESVi (ml/m^2^) | 31.7 (28.3-35.0) | 82.3 (57.6-95.1) | 0.122 |
| RA SVi (ml/m^2^) | 13.1 (7.3-18.8) | 14.6 (9.3-20.9) | 0.274 |
| RA Es (%) | 12.5 (10.5-14.6) | 12.2 (9.0-18.8) | 0.359 |
| RA Ee (%) | 4.4 (2.6-6.1) | 6.5 (4.5-9.4) | 0.549 |
| RA Ea (%) | 8.2 (7.9-8.5) | 5.8 (4.2-9.7) | 0.150 |
| **Tissue characterisation** | | | |
| Total LGE (3SD) (ml/m^2^) | 6.7 (3.2-10.1) | 4.9 (4.1-12.5) | 0.409 |
| T1 native septal (ms) | 1310.0 (1256.7-1318.0) | 1337.4 (1305.7-1372.5) | 0.234 |
| ECV total (%) | 25.3 (24.1-26.5) | 26.7 (24.4-29.4) | 0.062 |
| ECV corrected (%) | 24.8 (24.1-25.4) | 26.7 (24.4-29.4) | 0.062 |
| LV matrix volume (ml/m^2^) | 23.6 (17.8-29.4) | 25.6 (23.2-33.3) | 0.390 |
| LV cellular volume (ml/m^2^) | 71.3 (56.2-86.5) | 74.0 (70.2-77.5) | 0.964 |

Analysis of patient characteristics after dichotomisation at the median of EAT at 46.5ml/m^2^. Quantitative data are presented as median with interquartile ranges. Qualitative data are presented as n (%). The Mann-Whitney U test was used to test for significant differences. The asterisk indicates statistical significance. Ea: booster pump strain, EAT: epicardial adipose tissue, ECV: extra cellular volume, EDVi: end-diastolic volume index, Ee: conduit strain, Es: reservoir strain, ESVi: end-systolic volume index, GCS: global circumferential strain, GLS: global longitudinal strain, GRS: global radial strain, LA: left atrium, LGE: late gadolinium enhancement, LV: left ventricle, LVEF: left ventricular ejection fraction, RA: right atrium, SVi: stroke volume index

##### Supplementary Table 3: Characteristics of patients with low ejection fraction low-gradient aortic valve stenosis (type III)

| **Parameter** | **Low EAT volumes (*n*=11)** | **High EAT volumes (*n*=9)** | ***p*-value** |
| --- | --- | --- | --- |
| **Patient characteristics** | | | |
| Age at CMR (years) | 75.0 (71.8-80.3) | 80.0 (71.0-83.0) | 0.878 |
| Sex: female/male (*n*; %) | 3(27)/8(73) | 1(11)/8(89) | 0.369 |
| BMI (kg/m2) | 25.2 (22.4-28.0) | 30.8 (29.7-33.2) | 0.020* |
| Ejection fraction Simpson (%) | 37.6 (32.2-44.4) | 28.8 (25.0-43.7) | 0.849 |
| **Left ventricle** | | | |
| LVEF (%) | 38.5 (28.8-56.6) | 43.5 (31.1-58.5) | 0.210 |
| LV Mass (g/m^2^) | 93.8 (77.4-120.2) | 90.5 (79.1-125.4) | 0.790 |
| LV EDVi (ml/m^2^) | 110.6 (84.6-136.6) | 101.3 (82.3-135.6) | 0.569 |
| LV ESVi (ml/m^2^) | 67.1 (39.6-94.1) | 52.5 (35.9-93.0) | 0.569 |
| LV SVi (ml/m^2^) | 41.9 (36.2-50.0) | 48.7 (34.5-54.6) | 0.271 |
| LV GLS (%) | -13.1 (-17.2—10.3) | -18.8 (-20.2—7.2) | 0.730 |
| LV GCS (%) | -20.9 (-23.2—16.9) | -28.8 (-35.1—21.5) | 0.053 |
| LV GRS (%) | 39.4 (32.1-46.6) | 46.6 (37.4-64.5) | 0.133 |
| **Right ventricle** | | | |
| RVEF (%) | 44.8 (38.1-55.4) | 50.8 (47.6-51.8) | 0.382 |
| RV Mass (ml/m^2^) | 18.5 (16.2-22.3) | 15.0 (11.8-22.2) | 0.970 |
| RV EDVi (ml/m^2^) | 69.3 (61.4-75.8) | 68.2 (49.3-77.0) | 0.342 |
| RV ESVi (ml/m^2^) | 39.3 (25.3-46.6) | 32.4 (25.5-38.7) | 0.470 |
| RV SVi (ml/m^2^) | 31.3 (24.4-34.6) | 35.1 (23.8-38.7) | 0.732 |
| RV GLS (%) | -24.8 (-26.2—17.3) | -23.9 (-26.8—15.9) | 0.968 |
| **Left atrium** | | | |
| LA EDVi (ml/m^2^) | 65.1 (51.4-77.7) | 59.1 (50.0-69.9) | 0.248 |
| LA ESVi (ml/m^2^) | 48.8 (26.6-66.7) | 40.6 (30.4-58.4) | 0.477 |
| LA SVi (ml/m^2^) | 12.1 (10.2-23.5) | 18.0 (8.1-23.2) | 0.374 |
| LA Es (%) | 10.9 (8.0-19.0) | 11.0 (9.0-18.6) | 0.683 |
| LA Ee (%) | 5.1 (3.0-10.4) | 3.9 (1.7-8.5) | 0.514 |
| LA Ea (%) | 5.8 (3.4-12.8) | 7.9 (5.7-11.3) | 0.495 |
| **Right atrium** | | | |
| RA EDVi (ml/m^2^) | 46.3 (28.0-57.4) | 38.5 (37.2-58.8) | 0.457 |
| RA ESVi (ml/m^2^) | 34.5 (24.4-42.5) | 24.5 (21.0-40.9) | 0.741 |
| RA SVi (ml/m^2^) | 11.8 (3.7-14.9) | 14.7 (6.2-17.5) | 0.934 |
| RA Es (%) | 13.9 (11.8-15.1) | 16.5 (13.8-21.5) | 0.327 |
| RA Ee (%) | 6.5 (2.4-8.7) | 5.7 (3.8-7.7) | 0.806 |
| RA Ea (%) | 7.3 (4.9-10.3) | 11.9 (8.3-14.9) | 0.327 |
| **Tissue characterisation** | | | |
| Total LGE (3SD) (ml/m^2^) | 6.9 (2.8-14.9) | 14.6 (7.0-30.7) | 0.310 |
| T1 native septal (ms) | 1327.7 (1310.0-1362.9) | 1310.1 (1268.6-1365.0) | 0.366 |
| ECV total (%) | 29.7 (28.1-32.9) | 26.3 (25.0-29.3) | 0.203 |
| ECV corrected (%) | 26.8 (24.6-30.0) | 26.3 (25.0-28.3) | 0.685 |
| LV matrix volume (ml/m^2^) | 22.7 (13.3-34.6) | 22.2 (17.3-22.8) | 0.239 |
| LV cellular volume (ml/m^2^) | 61.0 (42.0-75.4) | 54.8 (48.3-66.0) | 0.425 |

Analysis of patient characteristics after dichotomisation at the median of EAT at 46.5ml/m^2^. Quantitative data are presented as median with interquartile ranges. Qualitative data are presented as n (%). The Mann-Whitney U test was used to test for significant differences. The asterisk indicates statistical significance. Ea: booster pump strain, EAT: epicardial adipose tissue, ECV: extra cellular volume, EDVi: end-diastolic volume index, Ee: conduit strain, Es: reservoir strain, ESVi: end-systolic volume index, GCS: global circumferential strain, GLS: global longitudinal strain, GRS: global radial strain, LA: left atrium, LGE: late gadolinium enhancement, LV: left ventricle, LVEF: left ventricular ejection fraction, RA: right atrium, SVi: stroke volume index

##### Supplementary Table 4: Characteristics of patients with paradoxical low flow low-gradient aortic valve stenosis (type IV)

| **Parameter** | **Low EAT volumes (*n*=18)** | **High EAT volumes (*n*=14)** | ***p*-value** |
| --- | --- | --- | --- |
| **Patient characteristics** | | | |
| Age at CMR (years) | 82.0 (80.0-85.0) | 78.0 (75.5-81.5) | 0.529 |
| Sex: female/male (*n*; %) | 12(67)/6(33) | 4(29)/10(71) | 0.033* |
| BMI (kg/m2) | 25.8 (24.2-30.8) | 29.8 (26.9-31.0) | 0.247 |
| Ejection fraction Simpson (%) | 58.0 (54.2-64.0) | 54.6 (51.0-58.2) | 0.565 |
| **Left ventricle** | | | |
| LVEF (%) | 67.0 (60.7-67.3) | 59.8 (45.9-68.3) | 0.430 |
| LV Mass (g/m^2^) | 62.4 (51.5-77.6) | 69.4 (62.2-74.9) | 0.618 |
| LV EDVi (ml/m^2^) | 70.7 (59.9-79.1) | 69.6 (63.9-78.2) | 0.678 |
| LV ESVi (ml/m^2^) | 23.4 (21.3-27.7) | 30.0 (19.7-43.5) | 0.561 |
| LV SVi (ml/m^2^) | 44.0 (38.9-51.4) | 34.5 (33.7-46.2) | 0.589 |
| LV GLS (%) | -22.8 (-26.1—19.1) | -19.8 (-24.1—17.2) | 0.355 |
| LV GCS (%) | -42.5 (-47.5—35.6) | -37.8 (-43.7—29.1) | 0.224 |
| LV GRS (%) | 79.5 (61.9-94.2) | 67.3 (44.3-81.7) | 0.120 |
| **Right ventricle** | | | |
| RVEF (%) | 51.4 (45.1-58.9) | 44.5 (42.9-58.5) | 0.835 |
| RV Mass (ml/m^2^) | 18.9 (12.1-22.3) | 20.5 (11.9-29.8) | 0.708 |
| RV EDVi (ml/m^2^) | 59.8 (55.9-76.0) | 78.4 (59.3-90.3) | 0.618 |
| RV ESVi (ml/m^2^) | 32.4 (24.2-36.1) | 41.2 (30.2-47.8) | 0.771 |
| RV SVi (ml/m^2^) | 34.8 (27.1-42.4) | 33.5 (29.1-45.3) | 0.934 |
| RV GLS (%) | -26.7 (-32.9—23.8) | -24.4 (-28.5—21.3) | 0.297 |
| **Left atrium** | | | |
| LA EDVi (ml/m^2^) | 50.0 (24.8-64.4) | 59.1 (40.4-76.0) | 0.096 |
| LA ESVi (ml/m^2^) | 35.9 (16.0-41.1) | 51.1 (24.5-66.6) | 0.038* |
| LA SVi (ml/m^2^) | 14.1 (11.7-20.4) | 9.0 (6.2-16.0) | 0.158 |
| LA Es (%) | 14.5 (10.9-22.4) | 9.4 (5.5-16.6) | 0.204 |
| LA Ee (%) | 5.0 (3.4-9.3) | 2.7 (1.4-6.2) | 0.393 |
| LA Ea (%) | 8.0 (6.2-10.5) | 5.6 (3.9-10.7) | 0.572 |
| **Right atrium** | | | |
| RA EDVi (ml/m^2^) | 39.9 (23.2-58.1) | 42.3 (25.9-70.8) | 0.561 |
| RA ESVi (ml/m^2^) | 30.1 (18.2-40.7) | 23.6 (17.9-52.6) | 0.803 |
| RA SVi (ml/m^2^) | 10.6 (5.0-15.1) | 9.7 (2.7-18.6) | 0.739 |
| RA Es (%) | 15.0 (12.3-24.4) | 14.4 (6.5-20.8) | 0.219 |
| RA Ee (%) | 6.8 (2.2-8.7) | 4.1 (1.2-9.1) | 0.812 |
| RA Ea (%) | 11.1 (7.5-13.4) | 8.1 (4.4-11.9) | 0.490 |
| **Tissue characterisation** | | | |
| Total LGE (3SD) (ml/m^2^) | 5.9 (3.8-12.4) | 6.3 (3.5-11.2) | 0.570 |
| T1 native septal (ms) | 1316.2 (1301.8-1337.0) | 1329.2 (1274.4-1362.9) | 0.944 |
| ECV total (%) | 27.7 (26.7-35.9) | 27.0 (24.9-28.8) | 0.293 |
| ECV corrected (%) | 26.7 (26.0-30.2) | 27.0 (25.0-27.9) | 0.921 |
| LV matrix volume (ml/m^2^) | 15.0 (11.2-19.4) | 15.7 (13.8-17.6) | 0.965 |
| LV cellular volume (ml/m^2^) | 38.3 (33.2-48.4) | 43.4 (38.6-47.2) | 0.930 |

Analysis of patient characteristics after dichotomisation at the median of EAT at 46.5ml/m^2^. Quantitative data are presented as median with interquartile ranges. Qualitative data are presented as n (%). The Mann-Whitney U test was used to test for significant differences. The asterisk indicates statistical significance. Ea: booster pump strain, EAT: epicardial adipose tissue, ECV: extra cellular volume, EDVi: end-diastolic volume index, Ee: conduit strain, Es: reservoir strain, ESVi: end-systolic volume index, GCS: global circumferential strain, GLS: global longitudinal strain, GRS: global radial strain, LA: left atrium, LGE: late gadolinium enhancement, LV: left ventricle, LVEF: left ventricular ejection fraction, RA: right atrium, SVi: stroke volume index
